# Steam-assisted respiratory muscle training may improve sleep quality in mild-to-moderate obstructive sleep apnea: a pilot polysomnography study

**DOI:** 10.1007/s44470-025-00036-w

**Published:** 2026-02-11

**Authors:** Usame Al-Rammahi, T. Soukka, V. Rimpilä, J. Malinen, RP. Happonen, A. Sovijärvi, U. Anttalainen

**Affiliations:** 1 Division of Surgery and Cancer Diseases, Department of Oral and Maxillofacial Surgery, Turku University Hospital, Kiinamyllynkatu 4-8, Turku, FI-20520 Finland; 2 Sleep Research Center, Department of Pulmonary Diseases and Clinical Allergology, University of Turku, Turku, Finland; 3 Department of Mathematics and Systems Analysis, Aalto University, Espoo, Finland; 4 Department of Clinical Physiology, University of Helsinki, Helsinki, Finland; 5 Division of Medicine, Department of Pulmonary Diseases, Turku University Hospital, Turku, Finland

**Keywords:** Respiratory exercises, Sleep quality, Polysomnography, Gender differences

## Abstract

**Study objectives:**

Obstructive sleep apnea (OSA) impairs sleep and respiration, and sub-optimal adherence to its gold-standard CPAP therapy compels development of alternative approaches. This study investigates the effects of steam-assisted respiratory muscle training (RMT) on polysomnographic (PSG) outcomes in patients with OSA.

**Methods:**

In a 12-week open-label prospective pilot study, 60 working participants with mild to moderate OSA underwent individualized inspiratory and expiratory resistance training with adjunctive steam inhalation. PSG was conducted pre- and post-intervention. Primary outcomes included changes in respiratory indices (AHI, ODI₃, CT₉₀) and sleep quality metrics (sleep efficiency, WASO). Statistical analyses included the Shapiro-Wilk normality test, Paired T, Welch, or Wilcoxon comparing visits, Wilson CIs reporting responders, Mann-Whitney and Fisher assessing associations with significance set at *p* < 0.05.

**Results:**

Of 60 participants, 33 completed the study. Primary outcomes–respiratory indices and sleep continuity metrics–remained unchanged (all *p* > 0.05). Secondary analyses showed reduced REM latency, increased REM duration, and fewer periodic limb movements and arousal-related events (all *p* < 0.05).

**Discussion:**

Steam-assisted RMT did not significantly alter respiratory or sleep continuity indices but was associated with modest changes in REM architecture and limb movements. These findings should be interpreted cautiously, as exploratory observations in a non-controlled pilot setting. Larger randomized, sham-controlled trials with objective adherence monitoring are warranted to confirm these preliminary results.

**Supplementary Information:**

The online version contains supplementary material available at https://doi.org/10.1007/s44470-025-00036-w.

## Brief summary

1. Study Rationale:
  Continuous positive airway pressure reduces upper-airway obstruction in obstructive sleep apnea but seldom normalises sleep architecture, and poor adherence limits its real-world impact. Respiratory-muscle training may strengthen airway dilator muscles, yet its effects on full polysomnography and periodic limb movements are largely unknown.
2. Study Impact:
  This pilot study was unable to demonstrate improvement in primary respiratory outcomes and thus cannot support RMT as an effective primary treatment for OSA at this stage. Nevertheless, exploratory findings regarding REM and PLM warrant further investigation in controlled trials.

## Introduction

Obstructive sleep apnea (OSA) is a prevalent sleep disorder characterized by recurrent upper airway obstruction during sleep, leading to intermittent hypoxia, sleep fragmentation, and increased sympathetic activation. Polysomnographic (PSG) hallmarks of OSA include an elevated apnea-hypopnea index (AHI), reduced rapid eye movement (REM) and slow-wave (N3) sleep, prolonged sleep latency, and frequent electroencephalogram (EEG) arousals [1]. Periodic limb movements during sleep (PLMS), involuntary, repetitive muscle contractions of the lower limbs, are frequently comorbid with OSA and contribute to disrupted sleep continuity. PLMS occur in 15–20% of individuals with OSA (PLMI ≥ 10–15 events/h) and are linked to reduced total sleep time, and poorer sleep efficiency [2]. Continuous positive airway pressure (CPAP) remains the first-line treatment for OSA, effectively lowering AHI and enhancing oxygen saturation [3]. However, CPAP demonstrates limited and inconsistent effects on sleep architecture and PLMS [4]. Notably, CPAP may unmask or even exacerbate PLMS in some patients, with studies reporting increased PLM indices post initiation, particularly among those without baseline PLMS [5]. Additionally, long term adherence to CPAP remains problematic, with up to 40% of users experiencing difficulties due to discomfort, device noise, or claustrophobia [6]. Alternative treatments, such as mandibular advancement devices (MADs) and upper airway surgeries, provide improvements in AHI and oxygen saturation but exert minimal influence on deeper sleep stages and arousal indices. For example, both CPAP and MADs produce only limited gains in REM and N3 sleep, without fully normalizing sleep architecture [7], while surgical options like uvulopalatopharyngoplasty yield variable results and often leave residual sleep fragmentation due to PLMS [8]. Given growing recognition of non anatomical OSA factors, including upper airway muscle tone, arousal threshold, and ventilatory instability, respiratory muscle training (RMT) has emerged as a promising non invasive therapeutic approach [9]. RMT may enhance respiratory strength and upper airway patency, potentially reducing airway collapsibility and improving ventilatory control [10]. Though under explored in OSA populations, evidence indicates that RMT may improve inspiratory muscle performance and mitigate sleep disordered breathing severity [11]. In our own 12-week pilot study, steam-assisted RMT was associated with improvements in patient-reported sleep apnea symptoms and pulmonary function in men and women with mild-to-moderate OSA, providing additional rationale to examine potential objective PSG changes following this intervention [12]. This pilot study investigates the use of WellO2^®^, a steam assisted RMT device that combines inspiratory and expiratory resistance training [13], in treatment of OSA patients. The predefined primary outcomes were respiratory indices (AHI, ODI₃, CT₉₀) and sleep continuity metrics (sleep efficiency, WASO), assessing whether RMT improves upper airway stability and overall sleep quality. Secondary exploratory outcomes included periodic limb movements and detailed sleep-stage architecture, evaluating potential ancillary effects of RMT on neuromuscular and arousal-related physiology.

## Methods

### Study design and participants

A pilot study with prospective clinical intervention study enrolled 60 adults aged 18–60 years with mild to moderate OSA (AHI of 5–29/h + typical OSA symptoms) were recruited from the Pulmonary Clinic at Turku University Hospital between May 2022 and August 2024. Their diagnoses were confirmed by home cardiorespiratory polygraphy in primary care clinics before referral to the Pulmonary clinic for treatment.

Final inclusion into the study was based on the first overnight polysomnography (PSG) confirming AHI < 30/h. The study excluded patients with a history of severe OSA (AHI ≥ 30/h), ongoing sleep apnea treatments, major oral or airway surgery, body mass index (BMI) > 40 kg/m², severe other pulmonary diseases (chronic obstructive pulmonary disease, asthma, pulmonary fibrosis, lung cancer), severe heart failure (NYHA 3–4), history of stroke or neuromuscular disease, unemployment, pregnancy, and disability to give their informed consent to the study.

The study protocol was approved by the Clinical Research Centre of the Turku University Hospital (T37/2021) and registered in ClinicalTrials.gov (NCT05320952). Ethical clearance was granted by the Southwest Finland Hospital District Ethics Committee (ETMK 31/1801/2021). The Finnish Medicines Agency (FIMEA) authorized the use of the WellO2^®^ device.

### Intervention

Participants completed a 12-week upper-airway respiratory muscle training program. Training consisted of inspiratory and expiratory counter-pressure breathing performed through a mouthpiece using a dedicated device that delivered adjustable steam and stepless adjustable resistance (settings 0–3, corresponding approximately to 15, 30, 65, and 120 cmH₂O, respectively). The device (WellO₂, WellO₂ Oy, Finland) combines exhalation into a heated water reservoir with inhalation through the same circuit, producing warm, humidified airflow and counter-pressure.

At baseline, maximal inspiratory and expiratory pressures (MIP/MEP) were measured with a MicroRPM^®^ device (CareFusion, UK). The target counter-pressure for each phase (inspiration or expiration) was then individually set to 30% of the measured MIP or MEP, respectively. Steam temperature in the training device was individually set to 55, 60, or 65 °C based on participant comfort.

Participants performed two sessions per day, each lasting up to 15 min, throughout the 12-week period. Sessions consisted of three sets of five deep, slow breathing against the device-generated counter-pressure via the mouthpiece, with a short rest between sets. The resistance setting (0–3) could be adjusted steplessly on the device to achieve the prescribed workload while maintaining a comfortable breathing pattern.

Proper technique and device operation were taught at initiation. Adherence support was provided via a mid-study follow-up call. Participants recorded use in paper diaries during three separate 2-week windows (beginning, middle, and end of the intervention), including session dates/times and device settings (temperature and resistance). Diaries were reviewed to assess adherence and protocol fidelity.

Usual care continued unchanged unless clinically indicated. Participants were instructed to stop a session if they experienced significant discomfort and to contact the study team; no other protocolized safety interventions were required. Device hygiene and handling followed the manufacturer’s general instructions.

### Measurements and assessments

Baseline evaluations included demographic data (age, gender, smoking status), anthropometric measurements (height, weight, BMI, waist and neck circumference), and medical history including medications. Overnight polysomnography (PSG) was conducted at baseline and after intervention to assess OSA severity.

Participants underwent single night PSG using NOX Medical NOX A1 device and Noxturnal Software System (versions 5.3 and 6.3). Recordings included electrocardiogram (ECG), electroencephalogram (EEG; channels: C3 A2, C4 A1, O1 A2, O2 A1, F3 A2, F4 A2), electrooculogram (EOG; two channels), and electromyogram (EMG; masseter, submental and anterior tibial electrodes).

Respiratory airflow was assessed using a nasal pressure cannula and oronasal thermistor for oral breathing detection. Thoracic and abdominal movements were monitored via respiratory inductance plethysmography, and oxygen saturation (SpO₂) via finger pulse oximetry [14].

Sleep stages were manually scored in 30 s epochs following American Academy of Sleep Medicine (AASM) guidelines (version 2.6) [15]. Obstructive apneas were defined as airflow cessation detected by nasal cannula and oral thermistor for ≥ 10 s, with continued respiratory effort. Obstructive hypopneas involved ≥ 30% airflow reduction lasting ≥ 10 s, associated with an arousal or ≥ 3% oxygen desaturation [15]. The AHI represented the hourly average of apneas and hypopneas.

Analyzed PSG parameters included sleep stage distribution (N1, N2, N3, REM), sleep efficiency, sleep latency, REM latency, and wake after sleep onset (WASO).

Additional parameters included arousal indices (total, respiratory related, PLM related, spontaneous), detailed respiratory indices (obstructive, mixed or central apneas and hypopneas), PLMS and related arousals, snoring percentage, airflow limitation, 3% oxygen desaturation index (ODI₃), mean and minimum SpO₂, cumulative duration with SpO₂ below 90% (CT₉₀), and heart rate (mean, maximum, minimum) [16].

Baseline and post intervention PSG values were paired within each participant. Improvement was recorded when the post value fell below baseline for event or time based indices (AHI, WASO, ODI₃, CT₉₀) or rose above baseline for favourable metrics (sleep efficiency, mean SpO₂, minimum SpO₂). The counts and participants who improved on each variable were tabulated and presented alongside pre specified responder thresholds from the OSA literature : the modified Sher/STAR rule for AHI (post AHI < 15/h and ≥ 50% reduction) [17]; ODI₃ ≥ 25% decrease or post ODI₃ <10/h; CT₉₀ ≥ 30% decrease or ≥ 5 min reduction [18]; sleep efficiency increase ≥ 5% or post intervention value ≥ 85% [19]; WASO decrease ≥ 20 min or ≥ 20% [20]; mean SpO₂ increase ≥ 1% [21]; and minimum SpO₂ increase ≥ 3% [22].

### Statistical methods

Statistical analyses were conducted using IBM SPSS Statistics (version 29.0.0.0). Normality was assessed using the Shapiro-Wilk test. Paired data comparisons between baseline and post-intervention were analyzed using Paired T-test for normally distributed variables or Wilcoxon Signed-Rank test when normality assumptions were violated. Welch’s T-test was applied exclusively to comparisons between independent groups exhibiting unequal variances.

Responder proportions and associated 95% confidence intervals were calculated using Wilson’s method. The associations between gender and binary responder status (responder/non-responder) were evaluated using Fisher’s exact test. Continuous responder metrics, if applicable, were compared by the Mann-Whitney U test. Bonferroni correction for multiple comparisons was applied, both unadjusted and Bonferroni-adjusted p-values are reported. Statistical significance after correction was set at *p* < 0.05.

## Results

### Study cohort and characteristics

Thirty-three participants (17 men, 16 women) out of 60 completed the 12-week study. Twenty-seven participants (45%) dropped out due to various reasons: 12 participants had baseline AHI-scores greater than 30/h, another 12 withdrew consent, and 3 were lost to follow-up. As shown in Table 1, excluded participants had a significantly higher body mass index, more severe overall and NREM-specific AHI scores, a greater total arousal index, and an elevated 3% oxygen desaturation index (ODI₃). Compared to men, the included women had significantly smaller neck circumference, and lower minimum SpO₂ and higher REM specific AHI.

**Table 1:** Included and excluded participants’ characteristics at baseline

Variable	Included Participants		Participants		
	Men	Women	Included	Excluded	*p*
Sample	17 (55.8%)	16 (44.2%)	33 (55%)	27 (45%)	0.4
Age	46 ± 8.5	50 ± 9.5	47.9 ± 9.1	46 ± 9.2	0.4
BMI (kg/m²)	28.3 ± 4.3	30.5 ± 4.7	28.6 ± 5.2	31 ± 2.9	**0.04**
Smoking (pack-years)	24 ± 17.8 (*n* = 5)	55.5 (*n* = 1)	29.3 ± 20.5 (*n* = 6)	18.6 ± 13.1 (*n* = 7)	0.3
Waist (cm)	103.1 ± 12	101.5 ± 15	102.3 ± 13.6	103 ± 11.4	0.8
Neck (cm)	42.2 ± 3*	37.9 ± 3*	38.9 ± 4.4	38.1 ± 2.5	0.1
Total sleep time (min)	415.6 ± 50.2	422.7 ± 45.3	418.6 ± 47.6	401.3 ± 72.4	0.3
REM sleep (%)	14.6 ± 5.4	13.8 ± 4.5	14.3 ± 5	14.7 ± 5.8	0.8
NREM sleep (%)	85.4 ± 21.6	86.2 ± 19.1	85.7 ± 18.6	85.3 ± 21.4	0.9
N1 (%)	6.6 ± 4.1	6.5 ± 4	6.6 ± 4	6.1 ± 4.2	0.6
N2 (%)	58.7 ± 8.7	55.8 ± 7.3	57.5 ± 8.1	60.8 ± 8.6	0.1
N3 (%)	20.1 ± 8.8	23.9 ± 7.8	21.7 ± 8.5	18.5 ± 8.6	0.1
Sleep effiency (%)	85.5 ± 9.5	84.5 ± 7.3	85.1 ± 8.5	82.4 ± 15.2	0.4
Sleep latency (min)	17.9 ± 13.2	25.6 ± 16.3	21.2 ± 14.8	21.3 ± 21.1	1
REM latency (min)	173.5 ± 72	165 ± 81.3	169.9 ± 74.9	184.8 ± 79.5	0.5
AHI_TOTAL_ (/h)	18.9 ± 6.4	20.5 ± 6.2	19.6 ± 6.3	30.1 ± 19.5	**0.01**
AHI_REM_ (/h)	32.6 ± 21.1*	49.8 ± 22.1*	39.9 ± 22.9	45.9 ± 26.6	0.4
AHI_NREM_ (/h)	17 ± 6.2	14.9 ± 5.2	16.1 ± 5.8	27.3 ± 21.2	**0.01**
ArI (/h)	10.4 ± 3.5	12.8 ± 3.9	11.4 ± 3.8	14.4 ± 6.8	**0.05**
ODI_3_ (/h)	17.4 ± 7.6	21.3 ± 6.2	19 ± 7.2	29.5 ± 18.2	**0.008**
Mean SpO_2_ (%)	93.2 ± 1.3	92.7 ± 1.2	93 ± 1.3	93.3 ± 1.5	0.4
Minimum SpO_2_ (%)	84.5 ± 3.5*	81.4 ± 4.3*	83 ± 4.1	81.8 ± 6.2	0.4
CT_90_ (%)	4.6 ± 8.8	6.5 ± 9.9	5.4 ± 9.2	4.3 ± 4.7	0.6

Values are given as mean ± standard deviation. Asterisk (*) indicates a statistically significant difference (*p* < 0.05) between included men and women, according to Welch’s T-Test. P-values presented in the last column represent comparisons between included and excluded participants, according to Welch’s T-test. *BMI* body mass index, *REM* rapid eye movement, *NREM* non-rapid eye movement, *N1/2/3* non-REM stage 1/2/3, *AHI* apnea-hypoapnea index, *ArI* All Arousals Index, *SpO₂* peripherial oxygen saturation, *ODI3* Oxygen Desaturation Index ≥ 3%, *CT90* cumulative time percentage with SpO₂ < 90%. Bold values indicate statistical significance (*p *< 0.05)

### Respiratory and oxygenation indices, and heart rate measures

No clinically or statistically significant changes were observed in respiratory or oxygenation parameters following RMT (Table 2, Supplement 1). All parameters remained essentially unchanged. No gender differences were found.

**Table 2:** Respiratory and heart-rate parameters at baseline and post-treatment

Respiratory and HR parameters	Baseline			Post-Intervention			Mean Difference (95% CI)			Total		
	Men	Women	Total	Men	Women	Total	Men	Women	Total	↓ % (*n*)	↑ % (*n*)	*p*
AHI_TOTAL_​	18.9 ± 6.4	20.5 ± 6.2	19.6 ± 6.3	18.8 ± 10.7	19.94 ± 8.4	19.3 ± 9.6	0.1 (−4.8, 4.6)	−0.6 (−5.7, 4.5)	−0.3 (−3.6, 3)	57.6% (19)	42.4% (14)	0.9
AHI_REM​_	32.6 ± 21.1	49.8 ± 22.1	39.9 ± 22.9	28.2 ± 21	50.9 ± 17.7	38.5 ± 22.7	−4.4 (−11.4, 2.7)	2.6 (−8.2, 13.4)	−1.4 (−7.3, 4.5)	54.5% (18)	45.5% (15)	0.6
AHI_NREM_​	17 ± 6.2	14.9 ± 5.2	16.1 ± 5.8	17.4 ± 12.9	13.4 ± 8.2	15.9 ± 11.1	0.4 (−4.7, 5.5)	−1.1 (−8.1, 4)	−0.2 (−3.7, 3.2)	54.5% (18)	45.5% (15)	0.9
OA_TOTAL​_	6.2 ± 5.2	5.5 ± 3.9	5.9 ± 4.6	7.7 ± 7	7 ± 4	7.4 ± 5.8	1.5 (−1.7, 4.6)	1.5 (−0.7, 3.7)	1.5 (−0.5, 3.4)	48.5% (16)	51.5% (17)	0.1
CA_TOTAL​_	0.4 ± 0.5	0.2 ± 0.3	0.3 ± 0.4	0.5 ± 0.7	0.3 ± 0.5	0.4 ± 0.6	0.09 (−0.3, 0.4)	0.1 (−0.2, 0.4)	0.1 (−0.4, 0.9)	36.4% (12)	42.4% (14)	0.4
MA_TOTAL​_	0.3 ± 0.6	0.04 ± 0.1	0.2 ± 0.5	0.7 ± 2.3	0.05 ± 0.1	0.4 ± 1.7	0.4 (−0.7, 1.6)	0.08 (−0.08, 0.09)	0.1 (−0.1, 0.3)	27.3% (9)	21.2% (7)	0.2
Snore (%)	41 ± 21	51.9 ± 28.6	46 ± 24	48 ± 22.6	52.4 ± 27	49.9 ± 24.3	7.1 (−3.8, 17.9)	0.5 (−7.6, 8.5)	4.3 (−2.6-11.1)	36.4% (12)	63.6% (21)	0.2
Flow limitation (%)	15.5 ± 8.6	16.4 ± 7.6	16 ± 8.1	14.6 ± 6.3	18.4 ± 11.3	16.2 ± 8.8	−1 (−3.9, 2)	2 (−2.9, 6.9)	0.3 (−2.3, 2.9)	39.4% (13)	54.5% (18)	0.8
ODI_3_ (h)	17.4 ± 7.6	21.3 ± 6.2	19 ± 7.2	19.5 ± 10.2	21.2 ± 8.2	20.2 ± 9.3	2.1 (−2.4, 6.7)	−0.1 (−4.7, 4.4)	1.2 (−1.9, 4.3)	45.5% (15)	54.5% (18)	0.4
Mean SpO_2​_ (%)	93.2 ± 1.3	92.7 ± 1.2	93 ± 1.3	93.4 ± 1.8	92.9 ± 1.2	93.2 ± 1.5	0.2 (−0.4, 0.8)	0.2 (−0.3, 0.7)	0.2 (−0.2, 0.6)	49.5% (17)	48.5% (16)	0.3
Minimum SpO_2​_ (%)	84.5 ± 3.5	81.4 ± 4.3	83 ± 4.1	84.8 ± 3.4	79.1 ± 7.6	82.4 ± 6.2	0.3 (−1.4, 1.9)	−2.3 (−5.6, 1)	−0.8 (−2.5, 0.8)	33.3% (11)	51.5% (17)	0.3
CT_90_ (%)	4.6 ± 8.8	6.5 ± 9.9	5.4 ± 9.2	6.3 ± 13.4	5.5 ± 8.6	5.9 ± 11.4	1.6 (−4.9, 8.2)	−1.1 (−6, 3.9)	0.5 (−3.6, 4.6)	51.5% (17)	42.4% (14)	0.9
Mean HR (min)	61.4 ± 8.0	67.2 ± 8.3	63.9 ± 8.5	61 ± 6.6	64.5 ± 8.6	62.5 ± 7.6	−0.4 (−2.2, 1.4)	−2.7 (−6.2, 0.8)	−1.4 (−3.1, 0.4)	54.5% (18)	39.4% (13)	0.1
Maximum HR (min)	98.3 ± 12.3	95.5 ± 17.6	94.2 ± 14.6	92.6 ± 13.5	88.6 ± 8.9	90.9 ± 11.7	−0.7 (−5.3, 3.9)	−6.9 (−17.7, 3.8)	−3.3 (−8.4, 1.7)	57.6% (19)	36.4% (12)	0.2

Values are presented as mean ± standard deviation (total *n* = 33; men = 17, women 16). P-values reflect within-subject comparisons between baseline and post-intervention values, calculated using the Paired T-Test (AHI_TOTAL_, AHI_REM_, AHI_NREM_, ODI_3_, Mean SpO₂) and the Wilcoxon Signed Rank Test (OA/CA/MA_TOTAL_, Snore, Flow limitation, CT_90_, Minimum SpO_2_, Mean/minimum/maximum HR). *AHI* apnea–hypopnea index, *REM* rapid eye movement sleep, *NREM* non-rapid eye movement sleep, *OA* obstructive apnea index, *CA* central apnea index, *MA* mixed apnea index, *ODI₃* 3% oxygen desaturation index, *SpO₂* oxygen saturation, *CT₉₀* cumulative percentage of time spent below 90% oxygen saturation, *HR* heart rate, *↓ (%)* percentage and number (n) of participants showing decrease, ↑ *(%)* percentage and number (n) of participants showing increase, *p* two-tailed statistical significance

Sympathetic outcomes displayed mean heart rate (HR) decreased in 55% of participants (18/33) and increased in 45% (15/33), yielding a mean change of −2.4 bpm (SD ± 1.6; 95% CI −6.2 to 1.4). Maximum HR fell by a mean of −4.8 bpm (SD ± 3.2; 95% CI −12.3 to 2.7), with 58% (19/33) improving and 42% (14/33) deteriorating. Minimum HR showed the smallest shift, improving in 40% (13/33) and declining in 60% (20/33), for a mean difference of −1.6 bpm (SD ± 1.1; 95% CI −4.2 to 1.0).

### Sleep continuity, architecture and periodic limb movements

REM-sleep latency shortened by 31 min after treatment (*p* = 0.04), whereas sleep latency, sleep efficiency, wake after sleep onset (WASO) and total sleep time remained unchanged (Table 3). The sleep stage distribution shifted (Table 4): the proportion of Stage N1 (*p* = 0.002) and REM sleep (*p* = 0.006) increased, while Stage N2 decreased (*p* = 0.03) and Stage N3 showed no significant change (*p* = 0.10). All indices of PLMS were significantly reduced following RMT (*p* < 0.05, Table 5). No statistically significant gender differences were found in sleep scoring, architecture and PLMS metrics.

**Table 3:** Sleep scoring at baseline and post-intervention

Sleep scoring	Baseline			Post-intervention			Mean Difference (95% CI)			Total		
	Men	Women	Total	Men	Women	Total	Men	Women	Total	Effect size	*p*	adj *p*
TST (min)	415.6 ± 50.2	422.7 ± 45.4	418.6 ± 47.6	401.5 ± 45.9	429.7 ± 43.3	413.4 ± 46.3	−14.1 (−41.5, 13.3)	7 (−29.1, 43.1)	−5.2 (−26.2, 15.9)	−0.13	0.5	1
Efficiency (%)	85.5 ± 9.5	84.5 ± 7.3	85.1 ± 8.5	81.3 ± 9.1	84 ± 9.9	82.4 ± 9.4	−4.3 (−10.3, 1.8)	−0.5 (−7.2, 6.2)	−2.7 (−7, 1.6)	−0.23	0.2	1
WASO (min)	67.3 ± 46.9	67.1 ± 30.3	67.2 ± 40.1	72.5 ± 38.6	54.7 ± 35.2	64.9 ± 37.7	5.1 (−9.9,30.1)	−12.4 (−37,12.2)	−2.3 (−19.4,14.8)	−0.02	0.9	1
Sleep latency (min)	17.9 ± 13.2	25.6 ± 16.3	21.2 ± 14.8	25.8 ± 46.5	27.4 ± 42.3	26.5 ± 44.1	7.9 (−5.5,31.2)	1.8 (−6.2,29.8)	5.3 (−1.7,22.2)	−0.23	0.2	1
REM latency (min)	173.5 ± 72	165 ± 81.3	170 ± 74.9	128.6 ± 45.1	152.7 ± 87.6	138.8 ± 66.4	−44.9 (−84.3,−5.4)	−12.3 (−50.7, 26.2)	−31 (−58.1, −4)	−0.4	**0.04**	0.2

Values are presented as mean ± standard deviation. *P*-values reflect within-subject comparisons between baseline and post-intervention values, calculated using the Wilcoxon Signed Rank Test. Bonferroni correction for multiple comparisons (m = 5) was applied; both unadjusted and Bonferroni-adjusted p-values (adj p) are reported. Statistical significance after correction was set at adj *p* < 0.05. Effect sizes were estimated using Rosenthal’s r. *TST* total sleep time, *WASO* wake after sleep onset, *REM* rapid eye movement, *p* two-tailed significance value. Bold values indicate statistical significance (*p* < 0.05)

**Table 4:** Sleep stages at baseline and post-intervention

Sleep stages	Baseline			Post-intervention			Mean Difference (95% CI)			Total		
	Men	Women	Total	Men	Women	Total	Men	Women	Total	Effect size	*p*	adj *p*
N1 (%)	6.3 ± 3.8	6.9 ± 4.4	6.6 ± 4	9.3 ± 5	12.9 ± 6.5	10.8 ± 5.9	3 (0.2,6)	5.9 (1,11)	4.2 (2,7)	−0.54	**0.002**	**0.008**
N2 (%)	57.5 ± 9	54.1 ± 13.5	56 ± 11.1	54 ± 7.6	54.2 ± 5.5	54.1 ± 6.7	−3.4 (−8,8)	0.14 (−8,9)	−1.9 (−6,3)	−0.4	**0.03**	0.12
N3 (%)	23 ± 9	21.7 ± 11.9	22.5 ± 10.1	21.5 ± 8.8	16.9 ± 6.4	19.5 ± 8.1	−1.6 (−5,2)	−4.8 (−10,1)	−2.9 (−6, 0.02)	−0.29	0.1	0.4
REM (%)	13.2 ± 5	15.7 ± 5.4	14.3 ± 5	16.7 ± 4.8	18.7 ± 6.6	17.5 ± 5.7	3.5 (1,6)	3 (−2,8)	3.2 (1,6)	−0.5	**0.006**	**0.024**

Values are presented as mean ± standard deviation. P-values reflect within-subject comparisons between baseline and post-intervention values, calculated using the Wilcoxon Signed Rank Test. Bonferroni correction for multiple comparisons (m = 4) was applied; both unadjusted and Bonferroni-adjusted p-values (adj p) are reported. Statistical significance after correction was set at adj *p* < 0.05. Effect sizes were estimated using Rosenthal’s r. *N1–N3* non-rapid eye movement (NREM) sleep stages, *REM* rapid eye movement sleep, *p* two-tailed significance value. Bold values indicate statistical significance (*p* < 0.05)

**Table 5:** Periodic limb movements in sleep at baseline and post-intervention

Periodic limb movement	Baseline			Post-intervention			Mean Difference (95% CI)			Total		
	Men	Women	Total	Men	Women	Total	Men	Women	Total	Effect size	*p*	adj *p*
PLMSc	126 ± 144	73 ± 112	103 ± 132	77 ± 118	58 ± 122	69 ± 118	−48 (−82, −14)	−15 (−53, 23)	−34 (−59, −10)	−0.54	**0.004**	**0.02**
PLMSi (/h)	18.7 ± 22	9.9 ± 15.2	15 ± 19.6	11.7 ± 18	7.9 ± 16.5	10.1 ± 17.2	−7 (−12, −2)	−2 (−8,4)	−5 (−8, −1)	−0.54	**0.003**	**0.01**
PLMSac	8 ± 10	11 ± 21	9 ± 15	4 ± 7	5 ± 10	4 ± 8	−4 (−11, 2)	−6 (−14,1)	−5 (−10, −0.4)	−0.49	**0.01**	**0.04**
PLMSai (/h)	1.2 ± 1.5	1.5 ± 2.8	1.3 ± 2.1	0.6 ± 1	0.6 ± 1.3	0.6 ± 1.1	−0.6 (−1.5 0.4)	−0.8 (−1.9, 0.2)	−0.7 (−1.3, −0.02)	−0.48	**0.02**	**0.08**

Values are presented as mean ± standard deviation. P-values reflect within-subject comparisons between baseline and post-intervention values, calculated using the Wilcoxon Signed Rank Test. Bonferroni correction for multiple comparisons (m = 4) was applied; both unadjusted and Bonferroni-adjusted p-values (adj p) are reported. Statistical significance after correction was set at adj *p* < 0.05. Effect sizes were estimated using Rosenthal’s r. *PLMSc* periodic limb movements during sleep (count), *PLMSi* PLM index (events per hour), *PLMSac* PLMs associated with arousals (count), *PLMSai* PLM arousal index (events per hour), *p* two-tailed significance value. Bold values indicate statistical significance (*p* < 0.05)

### High-responder analysis

Table 6 summarizes changes in key polysomnography (PSG) metrics post-intervention among participants who met the predefined literature-based criteria for clinically meaningful improvement (specified in Methods). Specifically, significant median reductions were observed in REM-specific AHI, total AHI, snoring time, ODI₃, and CT₉₀.

**Table 6:** Respiratory and oxygenation indices at baseline and post-intervention in participants with clinical improvements

Respiratory and oxygenation indices [m: w, *n*]	Baseline			Post-intervention			Mean Difference (95% CI)			Total		
	Men	Women	Total	Men	Women	Total	Men	Women	Total	Effect size	*p*	adj *p*
AHI_TOTAL_ (/h) [11:8, *n* = 19]	19.1 ± 8	22.3 ± 5	20.4 ± 7	12.8 ± 8	15.4 ± 5	13.9 ± 7	−6.3 (−9,−4)	−6.8 (−12,−2)	−6.5 (−9,−4)	−0.89	**0.002**	**0.01**
AHI_REM_ (/h) [11:6, *n* = 17]	36.4 ± 23	63.4 ± 24	46 ± 26	22.6 ± 22	49.9 ± 26	32.3 ± 26	−13.8 (−20,−8)	−13.5 (−22,−5)	−13.7 (−18,−9)	−0.5	**< 0.001**	**< 0.005**
AHI_NREM_ (/h) [11:7, *n* = 18]	16.2 ± 7	17.1 ± 4	16.5 ± 6	9.9 ± 7	8.5 ± 3	9.4 ± 6	−6.3 (−9,−4)	−8.5 (−14,−4)	−7.1 (−9,−5)	−1.13	**0.003**	**0.02**
Snore (%) [7:5, *n* = 12]	48.1 ± 16	54.2 ± 25	50.7 ± 19	36.1 ± 23	39.3 ± 20	37.4 ± 21	−12 (−20,−4)	−14.9 (−28,−2)	−13.2 (−19,−7)	−0.6	**< 0.001**	**< 0.005**
Flow limitation (%) [7:6, *n* = 13]	20.7 ± 11	18.7 ± 10	19.8 ± 10	14 ± 6	14.2 ± 10	14.1 ± 8	−6.7 (−13,−1)	−4.1 (−7,−1)	−5.6 (−9,−2)	−0.6	**0.004**	**0.02**
ODI_3_ (/h) [8:7, *n* = 15]	18.4 ± 6	23.4 ± 6	20.7 ± 6	12.2 ± 5	16.8 ± 5	14.3 ± 5	−6.2 (−9,−2)	−6.6 (−11,−2)	−6.4 (−9,−4)	−1.1	**< 0.001**	**< 0.004**
Mean SaO₂ (%) [7:8, *n* = 15]	93.2 ± 2	93 ± 1	92.1 ± 1	92.3 ± 2	92.6 ± 1	92.5 ± 2	−1 (−2,−1)	−0.4 (−1,−0.1)	−0.7 (−1,−0.4)	0.24	0.3	1
Minimum SpO_2_ (%) [8:8, *n* = 16]	85.4 ± 3	81.9 ± 5	83.5 ± 4	82.8 ± 3	76.7 ± 8	79.5 ± 7	−2.6 (−4,−2)	−5.2 (−11,−1)	−4 (−6,−2)	−0.66	**< 0.001**	**< 0.004**
CT_90_ (%) [8:7, *n* = 15]	7.4 ± 12	8.5 ± 13	7.9 ± 12	0.9 ± 1	3.5 ± 6	2.2 ± 4	−6.5 (−19,4)	−5 (−13,−2)	−5.7 (−12,−0.5)	−0.6	**0.02**	0.08

Values are presented as mean ± standard deviation. P-values reflect within-subject comparisons between baseline and post-intervention, calculated using the Paired T-Test (AHI_TOTAL_, AHI_REM_, AHI_NREM_, ODI_3_, Mean SpO₂) and Wilcoxon Signed Rank Test (Snore, Flow limitation, CT_90_, Minimum SpO_2_). Bonferroni correction for multiple comparisons (m = 5 (respiratory indices), and m = 4 (oxygenation)) was applied; both unadjusted and Bonferroni-adjusted p-values (adj p) are reported. Effect sizes were estimated using Rosenthal’s r (Snore, Flow limitation, CT_90_, Minimum SpO_2_) and Hedges’ g (AHI_TOTAL_, AHI_REM_, AHI_NREM_, ODI_3_, Mean SpO₂). *M: w* men and women ratio, *n* sample size, *AHI*_*REM*_ apnea–hypopnea index during REM sleep, *AHI*_*NREM*_ apnea–hypopnea index during NREM sleep, *ODI₃* 3% oxygen desaturation index, *SpO₂* oxygen saturation, *CT₉₀* cumulative percentage of time spent below 90% oxygen saturation. Bold values indicate statistical significance (*p* < 0.05)

Six participants (18%; 95% CI: 9–34%) met the modified Sher/STAR responder criteria, indicating a clinically meaningful AHI improvement. Separately, ten participants (30%; 95% CI: 17–47%) fulfilled the responder criterion based on ODI₃, while fourteen (42%; 95% CI: 27–59%) achieved the CT₉₀ threshold. Improvements in sleep continuity were common: seventeen participants (52%; 95% CI: 36–67%) increased sleep efficiency by ≥ 5% or normalized it to ≥ 85%, and twelve (36%; 95% CI: 22–53%) reduced wake-after-sleep-onset (WASO) by ≥ 20 min or ≥ 20%. Regarding oxygen saturation, nine participants (27%; 95% CI: 12–42%) increased mean overnight SpO₂ by ≥ 1%, while minimum SpO₂ increased by ≥ 3% in six participants (18%; 95% CI: 5–31%).

**Fig. 1:**
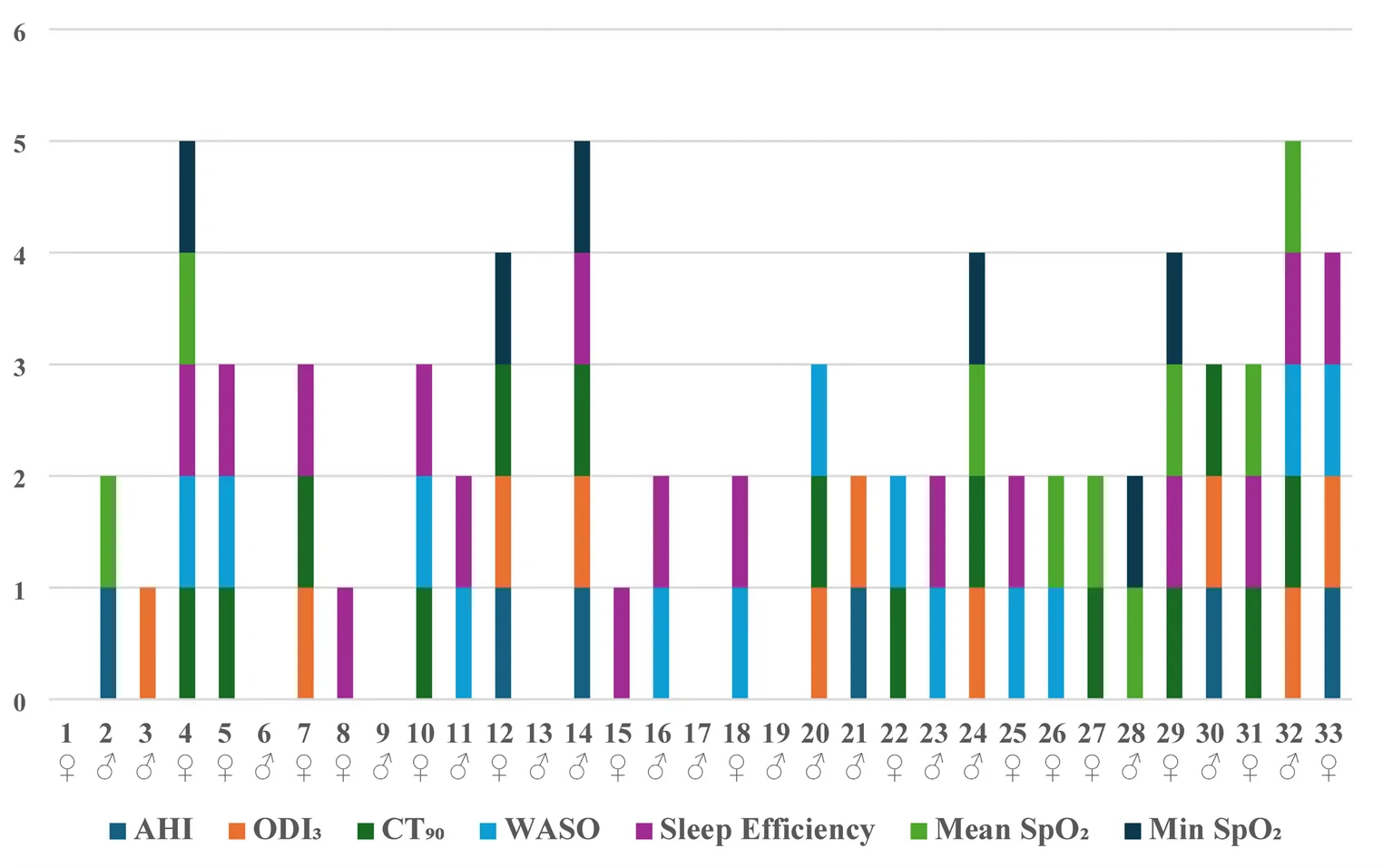
Stacked bar chart of participant-level changes across seven polysomnographic metrics after 12 weeks of steam-assisted respiratory muscle training. Each bar corresponds to a participant, segmented according to seven polysomnographic responder metrics: apnea-hypopnea index (AHI) [16], oxygen desaturation index (ODI₃) [17], cumulative time with oxygen saturation below 90% (CT₉₀) [17], sleep efficiency [18], wake after sleep onset (WASO) [19], mean oxygen saturation (Mean SpO₂) [20], and minimum oxygen saturation (Min SpO₂) [21]. Participant IDs and gender are displayed on the x-axis; horizontal gridlines indicate the cumulative number of metrics met

The median number of responder criteria achieved per participant was two (range: 0–5). The most frequent simultaneous improvements occurred in CT₉₀ and sleep efficiency (*n* = 8 participants; Fig. 1).

No significant differences emerged between men and women regarding the total number of responder criteria met (median test, Mann–Whitney U [*p* = 0.7]). Fisher’s exact tests for each individual PSG responder criterion (AHI, ODI₃, CT₉₀, WASO, sleep efficiency, mean SpO₂, minimum SpO₂) likewise revealed no gender differences (all *p* > 0.05).

## Discussion

This open-label pilot study of prospective clinical intervention of steam-assisted respiratory muscle training for 12 weeks suggests the intervention can improve certain aspects of sleep architecture and reduce PLM–related arousals in adults with mild to moderate OSA although respiratory indices did not decrease. In our previous study using the same steam-assisted RMT protocol, improvements were observed in patient-reported sleep apnoea symptoms together with modest gains in pulmonary function, supporting the clinical relevance of evaluating potential objective PSG correlates of these benefits [12]. 

At the cohort level, the primary outcomes–respiratory indices (AHI, snoring percentage, and flow limitation)–remained statistically unchanged, suggesting limited overall impact on upper airway stabilization. Similarly, cardiovascular and oxygenation parameters (heart rate metrics, ODI₃, mean and minimum SpO₂, and CT₉₀) showed no significant group-level changes. However, a subset of participants exhibiting clinically meaningful improvements demonstrated more pronounced gains across these measures. This interindividual variability highlights the heterogeneous nature of OSA treatment responses and the potential influence of anatomical factors, baseline severity, and adherence on therapeutic outcomes [9, 23]. Given the absence of a control group, these findings should be interpreted cautiously, as secondary changes in sleep architecture and limb-movement parameters may partly reflect night-to-night variability or acclimatization to repeated polysomnography recordings.In regards of sleep scoring, the observed 31 min reduction in REM latency indicates faster progression into REM sleep post intervention, potentially reflecting improved sleep regulation or respiratory stability [24]. In this cohort, baseline REM latency was substantially longer than typical normative values (mean ± SD: 170.0 ± 74.9 min), with 66.7% of participants exceeding 120 min. Following the intervention, mean REM latency decreased to 138.8 ± 66.4 min, yet 48.5% still remained above 120 min–both well beyond the expected normative range of 70–120 min [15]. While this reduction suggests a trend toward normalization, it should be interpreted cautiously, as factors such as physiological REM suppression or methodological factors can substantially affect REM onset timing. Typically, healthy adults enter REM sleep approximately 90 min after sleep onset; however, factors such as medications, circadian rhythms, and sleep fragmentation can significantly alter this timing, making REM latency a critical marker of sleep homeostasis [23]. Clinically, shorter REM latency may denote enhanced progression toward restorative sleep, crucial given REM sleep’s susceptibility to apnoeic disruptions [24]. Other sleep parameters, including total sleep time, latency, efficiency, and wake after sleep onset (WASO), showed no consistent changes, indicating that the intervention primarily affected sleep stage distribution rather than overall continuity.

In terms of sleep architecture, the proportion of Stage N1 and REM sleep increased, Stage N2 decreased, and Stage N3 remained unchanged, indicating a redistribution toward lighter and REM sleep stages. While stage N1 is typically considered light and less restorative, its increase may indicate a transitional adaptation to respiratory-muscle training, with airway stabilization achieved at the expense of deeper sleep onset. The concurrent shortening of REM latency and extension of REM duration imply a possible compensatory REM rebound, yet maladaptive fragmentation remains a plausible explanation. Increased REM may provide clinical benefits, particularly as REM suppression is common in OSA [25]. This REM increase may also reflect rebound sleep, as untreated OSA is associated with chronic REM deprivation, which often normalizes or overshoots upon effective intervention such as CPAP therapy [26, 27]. Previous research indicates RMT does not alter sleep stage distribution significantly [11]. Thus, the present findings of increased lighter sleep and REM stages may reflect individual variability in mild to moderate OSA responses and potential trade offs between sleep depth and respiratory stability.

At baseline, 33% of participants met the AASM criterion for elevated PLMS (PLMI ≥ 15 events/h), which lies toward the upper end of the prevalence range typically reported in OSA cohorts [2]. Prior study suggest that fluctuations < 10–15/h likely reflect night-to-night variability, whereas reductions ≥ 20–30 PLMS/h or ≥ 50%–particularly if accompanied by fewer arousals–may be clinically meaningful [2].

Moreover, PLM–related arousals notably decreased, correlating with substantial reductions in limb movement indices. This highlights differential responsiveness of arousal mechanisms to the intervention, underscoring the clinical importance of distinguishing arousal subtypes [28]. While CPAP is commonly reported to improve symptoms of restless legs syndrome and reduce limb movements in some patients with OSA, recent meta-analytic evidence indicates that CPAP may paradoxically increase the PLMI in certain populations, particularly those without baseline PLMS or with lower BMI [29]. Prior study demonstrated inconsistent PLM responses to CPAP, with some patients showing reductions while others experience unmasking or even increases in PLMI [5]. The observed PLM reduction with steam-assisted RMT may therefore reflect an alternative physiological pathway – possibly involving improved ventilatory efficiency and oxygen delivery – rather than the direct mechanical effects of upper airway splinting. One possible explanation for why breathing interventions might alleviate PLM relates to peripheral hypoxia. Salminen et al. demonstrated that peripheral hypoxia correlates with severity of RLS symptoms, suggesting that limb discomfort and movements may arise partly from impaired oxygenation in peripheral tissues [30]. This aligns well with observed reductions in CT₉₀ following the intervention.

A subset of participants demonstrated meaningful improvement in AHI, with approximately one fifth meeting the modified Sher/STAR responder criterion–fewer than typically reported for hypoglossal nerve stimulation [17] but comparable to outcomes of exercise-based OSA interventions [10, 11]. However, multi-night, PSG-validated data indicate substantial night-to-night variability, with wide limits of agreement (± 10–11 events/h) and about 20% reclassification when relying on a single night [30]. Accordingly, part of the apparent responder proportion may reflect spontaneous AHI fluctuation rather than a definitive intervention effect.

Even so, nearly half of participants exhibited improved oxygenation (shorter CT₉₀) and more than half showed enhanced sleep efficiency. These findings suggest that beneficial changes in desaturation depth and sleep continuity can occur independently of large AHI reductions, consistent with evidence that hypoxic-burden metrics predict cardiovascular outcomes more robustly than AHI alone [18]. Given the exploratory design, modest sample size, and absence of a control arm, these findings should be interpreted with appropriate caution. 

Overall, results emphasize the heterogeneity of mild to moderate OSA and support personalized approaches to treatment. Given that mild OSA effects vary substantially compared to severe forms with established cardiometabolic risks, further research with larger samples and extended follow up is needed to clarify response variability and refine intervention selection criteria [31].

A key strength of this study is its comprehensive in lab polysomnographic evaluation, which offers detailed insights into how RMT affects the sleep architecture in mild to moderate OSA. Subgroup analyses revealed heterogeneous treatment responses, highlighting the clinical variability of OSA, while the balanced enrollment of men and women provided preliminary data on potential gender specific outcomes. Moreover, the WellO2^®^ device uniquely integrates both inspiratory and expiratory resistance with warm, humid air inhalation, distinguishing it from other respiratory muscle training methods, which typically employ only inspiratory resistance and rarely incorporate steam. An additional benefit is the intentional incorporation of PSG derived outcome variables, as the literature reveals that a limited number of RMT trials, nine RCTs in systematic review [10] of which only four reported sleep efficiency and other polysomnographic metrics, and seven RCTs in a meta analysis [32] have utilized such variables, highlighting the scarcity of PSG based evidence in this domain [33].

The study has some limitations. Most importantly, because no control arm was included, observed changes may partly reflect regression to the mean, placebo effects, or selection bias rather than a true intervention effect. A control arm was not included because creating a sham device that would be indistinguishable to participants was not practically or ethically feasible for this device (credible shams for non-pharmacologic/device interventions are often difficult to design and maintain, and may expose participants to risks without prospect of direct benefit). Likewise, the study’s modest final sample size limited both statistical power and generalizability. The difference in results between home polygraphy and labbased polysomnography contributed to participant dropout. Excluding patients with severe OSA (AHI > 30) constrains applicability to those with higher disease burdens and may have introduced a selection bias toward individuals with better baseline respiratory stability or adherence potential. Furthermore, the lack of objective adherence monitoring for the WellO2^®^ device introduces uncertainty regarding treatment compliance; self-reported use may overestimate true adherence, potentially inflating the apparent intervention effect. Future studies should complete PSG eligibility screening before randomisation to minimize post-enrolment exclusions, and should incorporate digital adherence tracking and broader inclusion criteria would help validate and extend these findings. As a pilot, this study was not designed to provide a reliable treatment-effect estimate for powering a definitive RCT; therofore, sample-size calculations for a future trial should should be based on a clinically important target difference supported by prior evidence, using pilot data primarily to inform nuisance parameters and feasibility assumptions. Based on the observed variability in change scores among completers (*n* = 33), an indicative two-arm parallel RCT (1:1, two-sided α = 0.05) would require 110 participants to detect a statistically significant between-group difference.

Despite these limitations, findings support steam assisted RMT as a non invasive complement, for example for patients intolerant to CPAP. Future research should build upon recent findings demonstrating subjective sleep-quality improvements with steam-assisted respiratory muscle training by addressing key methodological limitations [12]. Randomized, sham-controlled trials with objective adherence monitoring are now essential to confirm causality and quantify the magnitude of physiological benefit. Beyond polysomnographic outcomes, studies should incorporate daytime functional measures–such as sleepiness, neurocognitive performance, and quality-of-life indices–to determine whether nocturnal improvements translate into meaningful daily benefits. Longitudinal designs with extended follow-up will help clarify whether changes in sleep architecture, including faster REM onset and reduced movement-related arousals, are transient adaptations or sustained effects. Finally, identification of baseline predictors of response could support more targeted implementation in mild-to-moderate OSA populations.

Steam-assisted respiratory muscle training significantly altered sleep architecture by reducing REM latency and increasing lighter (N1) and REM sleep in mild to moderate OSA. It also markedly decreased periodic limb movement–related arousals, indicating less limb-movement–related fragmentation. However, respiratory indices such as apnea–hypopnea events and snoring showed minimal overall changes, with notable improvements observed mainly in a subset of participants demonstrating clinically significant responses, highlighting the potential value of personalized treatment strategies. While larger trials are needed to confirm these preliminary findings, these pilot results do not yet demonstrate sustained benefit and should be interpreted with caution.

## Supplementary Information

Below is the link to the electronic supplementary material.

Supplementary Material 1

## Data Availability

De-identified data that support the findings are available from the corresponding author upon reasonable request, subject to institutional ethics approval.

## Abbreviations

AHI: Apnea–hypopnea index
BMI: Body-mass index
CPAP: Continuous positive airway pressure
CT_90_: Cumulative sleep time with SpO_2_ < 90%
ECG: Electrocardiogram
EEG: Electroencephalogram
EOG: Electro-oculogram
EMG: Electromyogram
FIMEA: Finnish Medicines Agency
HR: Heart rate
MAD: Mandibular advancement device
MIP and MEP: Maximal inspiratory and expiratory pressures
NREM: Non-rapid eye movement (sleep); N1, N2 and N3, stages 1, 2 and 3 of NREM sleep
NYHA: New York Heart Association functional class
ODI_3_: Oxygen-desaturation index using a 3% threshold
OSA: Obstructive sleep apnea
PLM: Periodic limb movement
PLMS: Periodic limb movements during sleep
PSG: Polysomnography
REM: Rapid eye movement (sleep)
RCT: Randomised controlled trial
RMT: Respiratory muscle training
SpO_2_: Peripheral oxyhaemoglobin saturation
UPPP: Uvulopalatopharyngoplasty
WASO: Wake after sleep
