# Correction to “Steam-assisted respiratory muscle training may improve sleep quality in mild-to-moderate obstructive sleep apnea: a pilot polysomnography study”

**DOI:** 10.1007/s44470-026-00182-9

**Published:** 2026-10-05

**Authors:** Usame Al-Rammahi, T. Soukka, V. Rimpilä, J. Malinen, RP. Happonen, A. Sovijärvi, U. Anttalainen

**Affiliations:** 1 Division of Surgery and Cancer Diseases, Department of Oral and Maxillofacial Surgery, Turku University Hospital, Kiinamyllynkatu 4-8, 20520 Turku, Finland; 2 Sleep Research Center, Department of Pulmonary Diseases and Clinical Allergology, University of Turku, Turku, Finland; 3 Department of Mathematics and Systems Analysis, Aalto University, Espoo, Finland; 4 Department of Clinical Physiology, University of Helsinki, Helsinki, Finland; 5 Division of Medicine, Department of Pulmonary Diseases, Turku University Hospital, Turku, Finland

**Correction to: Journal of Clinical Sleep Medicine**

https://doi.org/10.1007/s44470-025-00036-w

Corrections were identified post-publication by the authors of “Steam-assisted respiratory muscle training may improve sleep quality in mild-to-moderate obstructive sleep apnea: a pilot polysomnography study” [1].

Substantive report/model-output mismatch was found that affected the exploratory multiple-regression analyses presented in Table 7 and the related statements in the Abstract, Methods, Results, and Discussion sections of the paper.

The published model statistics and several coefficient entries could not be reproduced from the final dataset and are internally incompatible. Re-analysis produced consistent corrected results. Both corrected overall models remain statistically significant. However, BMI and waist circumference are strongly correlated (r = 0.870; VIF = 7.655 and 8.025), producing substantial multicollinearity and suppression. Consequently, the published statements concerning the independent relevance of the predictors and the interpretation of BMI as protective and waist circumference as a risk factor are not supported.

The corrections do not affect the participant data, sample size, intervention, paired pre–post analyses, primary respiratory or sleep-continuity outcomes, or the main conclusion of the article. It removes the secondary anthropometric predictor analysis and the interpretations derived from it.

The original article has been updated with the corrections listed below:

**Abstract**

• The final sentence of the Methods section has been revised from “Statistical analyses included the Shapiro–Wilk normality test, Paired T, Welch, or Wilcoxon comparing visits, Wilson CIs reporting responders, Mann–Whitney and Fisher assessing associations, regression predicting change, with significance set at p < 0.05.” to “Statistical analyses included the Shapiro–Wilk normality test, Paired T, Welch, or Wilcoxon comparing visits, Wilson CIs reporting responders, Mann–Whitney and Fisher assessing associations with significance set at p < 0.05.”

• The final sentence of the Results section has been deleted: “Regression analysis indicated that greater height and BMI were associated with fewer PLM, whereas larger waist circumference predicted more PLM.”

**Methods**

• In the second paragraph following the heading “Statistical methods,” the following sentence was deleted: “Linear regression analyses were performed to identify predictors of outcome changes.”

**Results**

• The following section was deleted:

**Determinants of PSG improvement**

Linear regression analyses were conducted to explore predictors of changes in significant PSG outcomes. Regression diagnostics confirmed acceptable assumptions (linearity, absence of significant collinearity after removing correlated variables, and residual normality).

As shown in Table 7, for periodic limb movements during sleep (ΔPLMS), the regression model was significant and explained 30% of variance (R2=0.50, adjusted R2=0.30, F = 2.7, p = 0.03). In the final adjusted model, significant predictors included height (cm; β=−1.9 PLMS units per cm increase; p = 0.02), BMI (kg/m2; β=−6.0 units per BMI unit increase; p = 0.02), and waist circumference (cm; β = 0.9 units increase per cm; p = 0.03). Due to known collinearity between BMI and waist circumference, exploratory analysis indicated stable coefficients when either BMI or waist circumference was excluded, confirming their independent relevance.

For PLMS-related arousals, the regression model was also statistically significant (R2=0.5, adjusted R2=0.3, F = 2.5, p = 0.04), accounting for 30% of variance. Increased height predicted fewer arousals, (β=−0.5 units per cm; p = 0.005), whereas weight (kg; β = 0.3 units per kg; p = 0.03) and waist circumference (cm; β = 0.2 units per cm; p = 0.01) were associated with increased arousals (Table 7).

In contrast, regression models for REM latency and percentages of N1, N2, and REM sleep did not yield statistically significant results (p > 0.05).

• Table 7 was deleted

**Figure:**
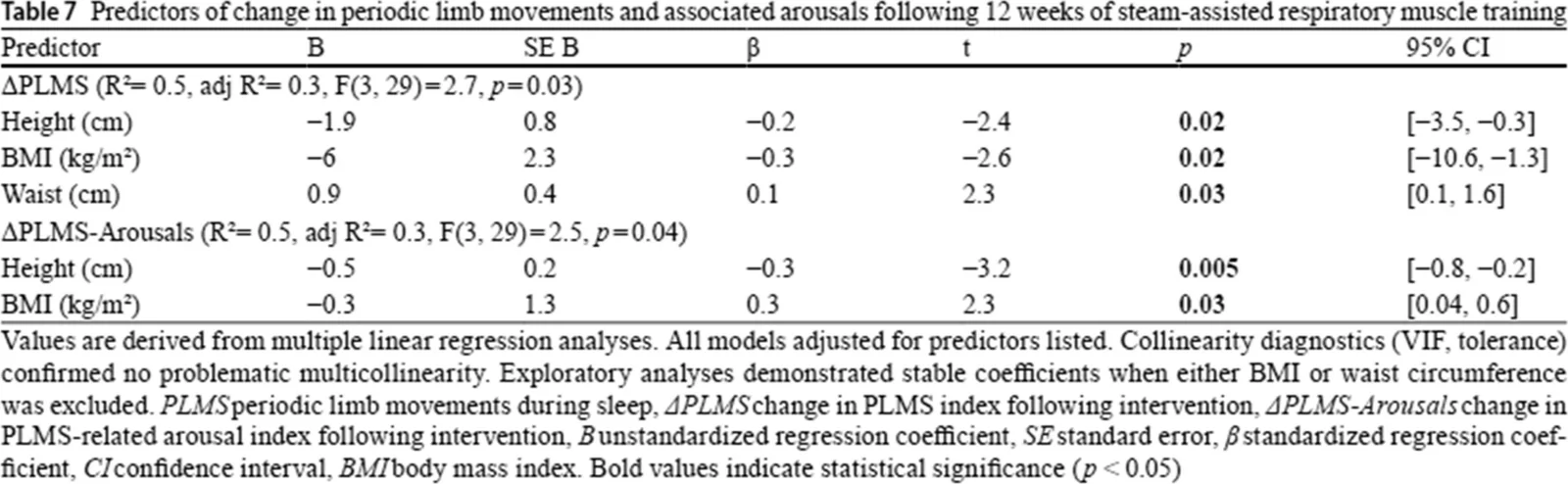

**Discussion**

• The sentence “This aligns well with observed reductions in CT₉₀ following the intervention, and also supports the regression findings, where greater waist circumference–potentially limiting diaphragmatic function and respiratory efficiency–predicted increased PLMS and related arousals.” was replaced with "This aligns well with observed reductions in CT₉₀ following the intervention.”

• The following paragraph was deleted:

Regression analyses further suggested that greater height and higher BMI acted as protective factors, potentially reducing limb movements and related arousals. Conversely, larger waist circumference consistently emerged as a risk factor, suggesting that central adiposity increases nocturnal limb activity and sleep disruption. However, these anthropometric predictors explained only about 30% of variance, indicating additional neurological, genetic, or lifestyle factors may be influential [26]. Parameters like REM latency showed minimal correlation with anthropometric measures, suggesting observed improvements primarily resulted from the intervention rather than participant characteristics. This highlights sleep physiology’s complexity, where multiple factors beyond anthropometry influence clinical outcomes [23].
