# Supplementary material for: Steam-assisted respiratory muscle training may improve sleep quality in mild-to-moderate obstructive sleep apnea: a pilot polysomnography study
Source: J Clin Sleep Med. 2026 Feb 11;22(1):32. doi: 10.1007/s44470-025-00036-w (PMC12995018; doi:10.1007/s44470-025-00036-w)
Supplement: Supplementary file 1 — Supplementary Material 1 [file 44470_2025_36_MOESM1_ESM.docx]

**Supplement 1.** Each point represents a participant’s baseline and post-intervention apnea-hypoapnea index. Lines connect within-subject values. Abbreviations: AHI, apnea–hypopnea index.
